# Helminthic Infection and Nutritional Studies among Orang Asli Children in Sekolah Kebangsaan Pos Legap, Perak

**DOI:** 10.1155/2016/1326085

**Published:** 2016-05-30

**Authors:** Weng Kin Wong, Phiaw Chong Foo, Mohamad Noor Mohamad Roze, Chau Dam Pim, Puvaneswari Subramaniam, Boon Huat Lim

**Affiliations:** ^1^School of Health Sciences, Universiti Sains Malaysia, 16150 Kubang Kerian, Malaysia; ^2^Tuberculosis and Leprosy Control Unit, Perak State Health Department, Ministry of Health, Jalan Panglima Bukit Gantang Wahab, 30590 Ipoh, Perak, Malaysia; ^3^Institute for Research in Molecular Medicine (INFORMM), Universiti Sains Malaysia, Health Campus, 16150 Kubang Kerian, Kelantan, Malaysia

## Abstract

*Background*. Orang Asli (aborigine) children are susceptible to soil-transmitted helminth (STH) infections due to their lifestyle and substandard sanitation system.* Objectives.* This study aimed to examine the helminthic and nutritional status of Orang Asli school children in Sekolah Kebangsaan Pos Legap, a remote primary school at Kuala Kangsar District in the state of Perak, Malaysia. In addition, the sensitivities of four STH stool examination techniques were also compared.* Methods.* Demography and anthropometry data were collected by one-to-one interview session. Collected stools were examined with four microscopy techniques, namely, direct wet mount, formalin ether concentration (FEC), Kato-Katz (KK), and Parasep*™*.* Results.* Anthropometry analysis showed that 78% (26/33) of children in SK Pos Legap were malnourished and 33% (11/33) of them were stunted. Stool examinations revealed almost all children (97%) were infected by either one of the three commonest STHs. FEC was the most sensitive method in detection of the three helminth species.* Conclusion*. This study revealed that STH infections and nutritional status still remain a health concern among the Orang Asli children. These communal problems could be effectively controlled by regular monitoring of STH infection loads, administration of effective antihelminthic drug regimen, and also implementation of effective school nutritional programs.

## 1. Introduction

Soil-transmitted helminth (STH) infections are infamous among children in rural areas such as aborigine settlements associated with substandard sanitation system and low socioeconomic status [1, 2]. The three common STH species that infect the school children are roundworm (*Ascaris lumbricoides*), whipworm (*Trichuris trichiura*), and hookworms. Often, two or more STH species are harbored by a child; and the infections are associated with poor growth, reduced physical activity, and impaired cognitive function and learning ability [3].

High intensity of STH infections in children showed negative impact on the nutritional status as infected children have decreased food intake, malabsorption, and poor food digestion [4]. This health problem remains unresolved due to the high recurrent infection percentage after treatment. In Malaysia, 50% of recurrent infections were reported to occur among aboriginal school children as early as 3 months after treatment, which increased up to 80% at 6 months after treatment, after a 3-day treatment regimen of 400 mg/daily of albendazole tablets [1, 5]. Therefore, in high STH endemic areas, it is more pertinent to determine the load of STH infections than merely identifying STH cases. Regular STH monitoring and deworming exercises are necessary to reduce the load of infections, which can effectively minimize eggs deposited into the environment and subsequently decrease the probability of recurrent infections.

There are a number of useful microscopy stool examination techniques. Direct wet mount (DWM) is routinely used for qualitative detection of STH ova or larvae [6]. World Health Organization (WHO) recommended Kato-Katz (KK) technique for quantitative examination due its simplicity and capability to determine load of the infections. However, formalin ether concentration (FEC) technique is the most sensitive laboratory technique for detection and quantification of STH [7]. Recently, Parasep (DiaSys, UK) was introduced to make FEC more user-friendly [8].

This study aimed to explore the incidence and load of STH infections as well as the nutritional status of Orang Asli school children in Pos Legap settlement at Sungai Siput District in the state of Perak, Malaysia. In addition, the sensitivities of DWM, KK, FEC, and Parasep were compared.

## 2. Methods

### 2.1. Subject and Study Area

Pos Legap Orang Asli settlement is built under the Forth Malaysia Plan: Orang Asli Reclustering Plan (*Rancangan Pengumpulan Semula*,* RPS*) carried out by the Department of Orang Asli Affairs (JAKOA) under the purview Ministry of Rural and Regional Development [9]. The place is situated 248 km from Kuala Lumpur and 20 km to the nearest Sungai Siput town. The idea of RPS was to relocate all the Orang Asli communities from the rural settlements to the peripheral urban areas to facilitate access to better facilities especially those related to education and health care. Their educational level and socioeconomic status have gradually improved while preserving their unique culture [10]. In this study, school children of standards 1 to 3 were recruited from Sekolah Kebangsaan Pos Legap, the only primary school in that settlement. The estimated 200 children registered in the school were from the Temiar tribe. However, high absenteeism was common in schools located at Orang Asli settlements [11, 12].

### 2.2. Questionnaire and Sample Collection

Data and sample were collected during a five-day visit to Sekolah Kebangsaan Pos Legap. A questionnaire session was conducted through one-to-one interview. The questionnaire comprised two parts: (a) demography data and (b) daily practices of the school children evaluated based on 4-point Likert scale ((1) Never, (2) Seldom, (3) Usually, and (4) Always). Each child was then given a biohazard zipper bag containing stool container and spatula. They were requested to return the stool sample the following day. The stool samples were kept in ice box and transported to the laboratory for examination. Anthropometric data such as body weight and height were obtained in order to get insight into the children nutritional status and growth. They were weighed wearing school uniforms, without belt and shoes and with empty pockets using calibrated Weighting Scale 762 (SECA, Germany). The height was measured using Bodymeter 206 (SECA, Germany). Both weight and height of subject were recorded to the nearest reading of 0.1 cm and 0.1 Kg, respectively.

### 2.3. Stool Examination

Each stool sample was aliquoted for direct examination using DWM and KK. The remaining stool sample was fixed in 10% formalin solution prior to microscopic examination using FEC and Parasep (DiaSys, UK). For microscopic examination, the stool sample on the glass slide covered by either glass cover slip or cellophane membrane was examined under the 100x magnification. In DWM [7], a drop of normal saline and Lugol iodine was first dropped on glass slide, separately. A spike of stool sample was mixed with the droplets using applicator stick. A cover slip was put onto each mixed sample and examined under microscope.

FEC [7] was performed by mixing one gram of stool with 5 mL of 10% formalin and straining through 1 mm stainless steel sieve. The suspension was added to 15 mL centrifuge tube and centrifuged at 440 ×g for 2 min. The supernatant was discarded and the pellet was washed with 10% formalin for another two times. After washing, the pellet was mixed with 5 mL of 10% formalin. About 3 mL of ether solution was added to the suspension and shook vigorously to mix the suspension. The suspension was centrifuged at 440 ×g for 2 min. Four layers of suspension were formed, that is, ether layer at the top most, followed by fatty plug, 10% formalin, and sample pellet. The upper three layers were aspirated off with disposable Pasteur pipette. The fatty plug was loosened with applicator stick and then the upper three layers were aspirated off using disposable Pasteur pipette; and about 500 *μ*L of 10% formalin remained in the tube. The concentrated sample was mixed with the formalin solution followed by microscopic examination.

Parasep was performed according to the manufacturer's protocol (DiaSys Europe Ltd., Berkshire, United Kingdom). Briefly, a scoop of stool sample was picked using Parasep spoon and mixed in 2.4 mL of 10% formalin solution added with a drop of Trition-X 100 surfactant, in the mixing compartment. The mixing compartment was weighed before and after adding the stool sample to the mixing compartment for estimation of stool sample weight. After that, the mixture was mixed and emulsified by vortexing. The tube was then inverted and centrifuged at 1000 ×g for 3 minutes. The sample was then filtered into the filter compartment. Four layers were formed in the filter compartment, that is, ether layer, fatty plug, 10% formalin, and sample pellet, from the top of the filter compartment. The fatty plug was loosened with applicator stick and then the upper three layers were aspirated off with disposable Pasteur pipette, and about 500 *μ*L of 10% formalin remained in the tube. The concentrated sample was mixed with the formalin solution and followed by microscopic examination.

Kato-Katz [13] was performed by passing the stool sample through 0.1 mm screen. About 40 mg of sieved stool sample was added in the hole of a Kato-Katz plastic template prefixed on a glass slide. The template was lifted. A drop of malachite green solution was used to stain the stool sample on the glass slide. Cellophane membrane was used to cover the stained sample, which was then observed under microscope.

### 2.4. Data Analysis

For anthropometric analysis, height-for-age* Z*-score was used as an indicator for stunting (chronic malnutrition) or short stature for the child's age. Weight-for-age* Z*-score was used as the general indicator for child general protein-energy malnutrition (underweight) for his/her age. BMI-for-age* Z*-score was used to determine whether the child is too thin for his/her height. For this study, the severity of the malnutrition was graded based on the median values in the reference chart for analysis of nutritional status among children and adolescents 5–19 years according to WHO 2007 growth reference [14]. Children who have* Z*-scores below −2 standard deviations (SD) of the NCHS reference population median values were considered to be significantly malnourished/stunted while* Z*-scores between −2 SD and −1 SD were considered to be mildly malnourished/stunted. For BMI-for-age,* Z*-scores < −2 SD were considered to be thin and* Z*-scores < −3 SD were considered to be severely thin.

The load of infection for* A. lumbricoides* was characterized according to WHO technical report, that is, low (<5000 eggs per gram), moderate (5000 ≤ *X* < 50000 eggs per gram), and high (≥50000 eggs per gram). For* T. trichiura* infections, the load of infection was characterized as low (<1000 eggs per gram), moderate (1000 ≤ *X* < 10000 eggs per gram), and high (≥10000 eggs per gram), whereas hookworm infection was characterized as low (<2000 eggs per gram), moderate (2000 ≤ *X* < 4000 eggs per gram), and high (≥4000 eggs per gram) [15].

### 2.5. Ethical Consideration

The proposal of this study was approved by Medical Research Ethics Committee, Malaysia Ministry of Health (Ref. number: NMRR-11-664-9826) and also Ministry of Education, Malaysia (Ref. number: KP(BPPDP)603/5/JLD.14(05)). School children were recruited as participants after written informed consents were obtained from the school principal and also the children who stayed in the school hostel. The analysis report of STH-infected children was submitted to Dr. Puvaneswari Subramaniam at the Perak State Health Department, who subsequently ensured that the Sungai Siput District Medical Team and the headmaster of Sekolah Kebangsaan Pos Legap perform the appropriate actions, such as treating the children and their respective family members with albendazole, as well as advising the children and their parents on the importance of hand hygiene and use of footwear through health education.

## 3. Results

### 3.1. General Characteristics

A total of 33 Orang Asli school children completed the questionnaire and provided the stool samples (Table 1). Their ages ranged between 7 and 9 years and comprised 42% girls and 58% boys. More than 85% of their parents never attended school and the highest education level attended by their parents was at secondary school level. Most of their parents were farmers, rubber tappers, and hunters in the forest. Their brick houses were built by the Malaysian government. The water source was from river and some of the houses were equipped with direct piping system. There was no modern sanitation system in the Orang Asli settlements.

### 3.2. Status of Parasitic Infection and Nutritional Status

A total of 33 stool samples were examined using the four stool examination techniques (Table 2). Almost all stool samples (97%) were found to be positive for either one of the STH species. About 75% of the positive stool samples were found to be mixed infections. The types of mixed infections were ALO + TTO (22%), TTO + HWO (25%), and ALO + TTO + HWO (28%). The positive percentage of ALO infection among the 33 school children was 55%, whereas positive percentage of TTO and HWO infections was 88% and 52%, respectively. Among the 18 ALO positive samples, 72% were found to be of low intensity, 22% of moderate intensity, and 6% of high intensity infections. Twenty-nine TTO positive samples were of low intensity infection (93%), while the remaining cases were of moderate intensity infection. All HWO positive samples were of low intensity infection. The anthropometry analysis showed that 78% of the Orang Asli school children were malnourished; 33% of them were stunted and about 72% fell within the range of mildly thin to severely thin. Interestingly, intestinal protozoa were not observed in this study.

### 3.3. Comparison of Sensitivities of Four Different Stool Examination Techniques

**Figure 1:**
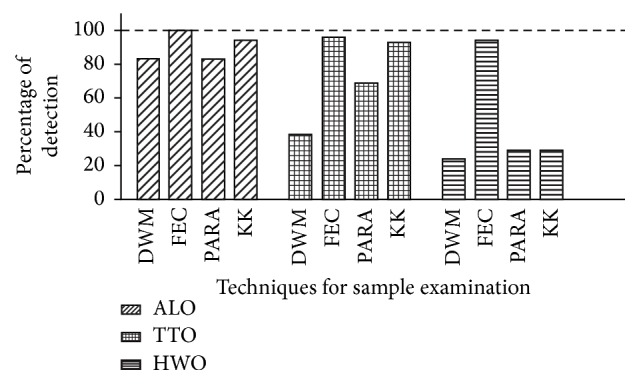
Sensitivities of four STH examination techniques.

Figure 1 shows the result of STH species detection by using four different types of stool examination techniques. For detection of ALO, all four techniques showed sensitivities of above 80%. FEC and KK detected more ALO cases than the other two techniques, that is, 100% and 94%, respectively. Similarly, in detection of TTO, FEC and KK showed higher detection percentages, that is, 97% and 93%, respectively, followed by Parasep and DWM with only 69% and 38%, respectively. In the detection of HWO, FEC showed the highest sensitivity of 94%, followed by Parasep, KK, and DWM with detection percentages as low as 29%, 29%, and 24%, respectively.

### 3.4. Personal Hygiene Practice

According to the data compiled from the one-to-one interview concerning their personal hygiene practice (Table 3), the school children usually washed their hands before eating and after going to the toilet. They usually washed hands with soap whenever soap is available. The children also usually washed vegetables and/or fruits before eating them. Most of the students usually wore shoes outside the house and rarely wore them inside the house. Due to the proper sanitation system in the school hostel, the students usually defecated or urinated in the school toilets and rarely in open space. They usually drank boiled water and also ate with their bare hands. Animals were rarely found in their houses. They also rarely wash their feet before entering the house due to the limited water piping facilities.

## 4. Discussion

According to JAKOA annual report [10], the Malaysian government has provided medical services to the Orang Asli population since 1970 through mobile clinic services at their settlements. One of the health services is the deworming program which targeted 2–12-year-old children every year and also included adults with intestinal problems, with single- and two-dosage 400 mg albendazole, respectively. This study showed that the majority of STH infections among school children were maintained at low intensity level; however, two-thirds of the school children were still malnourished and thin although Malaysia Ministry of Education and JAKOA have been providing healthy food to these children under the school nutrition program. The poor nutritional status is probably due to STH infections. This negative association was also observed in children suffering from ascariasis and trichuriasis, who experienced reduced food intake, impaired digestion, malabsorption, and poor growth rate [8]. Hence, it is important to reduce the load of infections to minimize the eggs from being deposited to the environment that probably caused the persistent high rate of STH infections among the Orang Asli school children [16].

In comparing the four stool examination techniques for detection of ALO, TTO, and HWO, FEC showed the highest sensitivity followed by KK, Parasep, and DWM. Generally, FEC, KK, and Parasep showed high sensitivities (>90%) in detection of the presence of either STH. KK and Parasep detected less HWO as compared to FEC as the latter could process bigger quantity of stool sample in a single test.

Coherent with previous findings, the predominant STH species found among Orang Asli school children in Pos Legap were also* T. Trichiura*, followed by* A. lumbricoides* and hookworms [1]. From the quantitative stool examination using FEC, most of the children were infected with low to moderate intensity of STH. The nutritional status of the Orang Asli school children was poor, as about one-third of them were found to be stunted while the rest were thin and malnourished.

## 5. Conclusion

STH infections still prevail among Orang Asli school children staying in peripheral urban areas such as Pos Legap settlement. The Malaysian government has gradually improved the socioeconomic status, educational standard, and health facilities in the Orang Asli settlements but much more could still be done. Therefore, effective antihelminthic drug regimen has to be administered; and improved nutritional programs have to be provided to ensure Orang Asli children can have normal growth and development. This will enable the future Orang Asli generations to be more competitive in the future.

## Figures and Tables

**Table 1:** Demography data of school children in this study.

Variables	*N* (%)
Age	
7	7 (21%)
8	7 (21%)
9	19 (58%)
Gender	
Female	14 (42%)
Male	19 (58%)
Mother's education	
Never go to school	28 (85%)
Primary school	5 (5%)
Father's education	
Never attend school	30 (91%)
Primary school	1 (3%)
Secondary school	2 (6%)
Water source	
River	10 (33%)
Pipe	23 (67%)
Toilet	
In the house	12 (36%)
Outside of the house	21 (64%)

**Table 2:** Nutritional and STH infection status.

Variable	*N* (%)
Weight-for-age	
Nonmalnourished	7/33 (21%)
Mild malnourished	12/33 (36%)
Moderate malnourished	10/33 (30%)
Severe malnourished	4/33 (12%)
Height-for-age	
Nonstunted	22/33 (67%)
Mild stunted	11/33 (33%)
BMI-for-age	
Nonthinness	9/33 (27%)
Mild thinness	14/33 (42%)
Severe thinness	10/33 (30%)
Parasitic infection status	
Positive by either one	32/33 (97%)
DWM	22/32 (69%)
FEC	32/32 (100%)
Parasep	30/32 (94%)
KK	31/32 (97%)
Types of infection	
Single	8/32 (25%)
ALO	3/32 (9%)
TTO	5/32 (16%)
HWO	—
Mixed	24/32 (75%)
ALO + TTO	7/32 (22%)
TTO + HWO	8/32 (25%)
ALO + TTO + HWO	9/32 (28%)
Load of infection	
ALO	18/33 (55%)
Low	13/18 (72%)
Moderate	4/18 (22%)
High	1/18 (6%)
TTO	29/33 (88%)
Low	27/29 (93%)
Moderate	2/29 (7%)
HWO	17/33 (52%)
Low	17/17 (100%)

**Table 3:** Daily practices of Orang Asli school children.

Daily practice	Mean score ± SD
Wash hand with soap before eating	2.61 ± 0.933
Wash hand without soap before eating	2.85 ± 0.712
Wash hand with soap after going to the toilet	2.61 ± 0.864
Wash hand without soap after going to the toilet	2.67 ± 0.777
Wash vegetables and/or fruits before eating	2.79 ± 0.781
Wear shoes outside the house	2.97 ± 0.770
Wear shoes inside the house	1.58 ± 0.830
Defecate or urinate in the toilet	2.94 ± 0.827
Defecate or urinate at open space	2.18 ± 0.846
Drink boiled water	2.73 ± 0.761
Eat using bare hand (without using fork and spoon)	2.88 ± 0.545
Keep animal inside the house	2.24 ± 1.062
Wash feet before entering the house	2.36 ± 0.783
